# Reinvigorating drug development around NGF signaling for pain

**DOI:** 10.1172/JCI189029

**Published:** 2025-02-17

**Authors:** Andi Wangzhou, Theodore J. Price

**Affiliations:** University of Texas at Dallas, Center for Advanced Pain Studies and Department of Neuroscience, Richardson, Texas, USA.

## Abstract

Nerve growth factor (NGF) signaling is a clinically validated target for the treatment of several prevalent types of chronic pain; however, addressing safety concerns remain a key challenge. In this issue of the *JCI*, Peach et al. take a major step forward in this area by deciphering complexities in the signaling of the NGF receptor TrkA, finding that neuropilin 1 (NRP1) acted as a coreceptor for NGF actions at TrkA and the receptor complex required scaffolding from GIPC1. Using a mix of techniques, including animal behavioral models, electrophysiology on mouse and human dorsal root ganglion (DRG) neurons, and elegant biochemical pharmacology, the authors demonstrated that this therapeutic target might more safely manipulate NGF signaling to achieve pain alleviation. While there are still important questions to answer, the innovative work paves the way for the development of nonopioid pain therapeutics.

## The NGF pathway in pain therapeutics

Chronic pain, afflicting at least hundreds of millions globally, remains inadequately addressed by conventional analgesics such as NSAIDs and opioids due to limited efficacy and severe side effects. One of the most promising nonopioid therapeutics to emerge in the past two decades was the monoclonal antibody-sequestering nerve growth factor (NGF) tanezumab, which showed efficacy in clinical trials for osteoarthritis (1), low back pain (2), neuropathic pain (3), and cancer pain (4). The development of tanezumab was based on decades of research starting with the pioneering discovery of NGF by Rita Levi-Montalcini (5) and the later discovery that loss of function mutations in NGF signaling lead to congenital insensitivity to pain (6). Further insightful work of Stephen McMahon and others, showed that NGF sensitizes adult tissue damage and injury-sensing neurons, called nociceptors, in the dorsal root ganglion (DRG), thereby, promoting pain (7). Despite its efficacy for pain, tanezumab was ultimately not approved by the FDA because of safety issues related to the risk of rapidly progressing osteoarthritis in 3% to 6% of patients, depending on tanezumab dose (8). Therefore, NGF signaling is a clinically proven target for nonopioid pain therapeutic development, but targeting NGF itself has safety liabilities that likely preclude approval of NGF sequestering biologics. These outcomes underscore the necessity of refining therapeutic strategies to harness the NGF pathway’s potential while circumventing adverse effects.

NGF primarily exerts its effects through two receptors, TrkA and p75, both playing distinct roles in neuronal development, pain sensitization, and apoptosis regulation. While p75 is widely expressed in adult humans, TrkA (encoded by the *NTRK1* gene) has a more restricted expression pattern with relative enrichment in the DRG (9) making this receptor an attractive target. In the human DRG, TrkA is expressed by most presumptive nociceptors into adulthood (10, 11) and the receptor is clearly important for the development of these neurons based on genetic findings (6). Another side effect noted in tanezumab trials was minor loss of sensation and tingling in extremities (2, 12).

In this issue of the *JCI*, Peach et al. highlight a critical advancement in this domain by introducing neuropilin 1 (NRP1) as a coreceptor that modulates NGF/TrkA signaling (13). This discovery not only elucidates key mechanisms underlying NGF-mediated pain pathways, but also presents NRP1 and associated cofactors such as GIPC1 as alternative, and potentially safer, therapeutic targets. The study provides preclinical behavioral, electrophysiological, and biochemical evidence for the efficacy of NRP1 inhibitors in mitigating NGF-induced nociception and nociceptor sensitization, offering an exciting possibility for nonopioid pain management through targeting a clinically validated signaling pathway.

## The NRP1 coreceptor in NGF/TrkA Signaling

**Figure 1:**
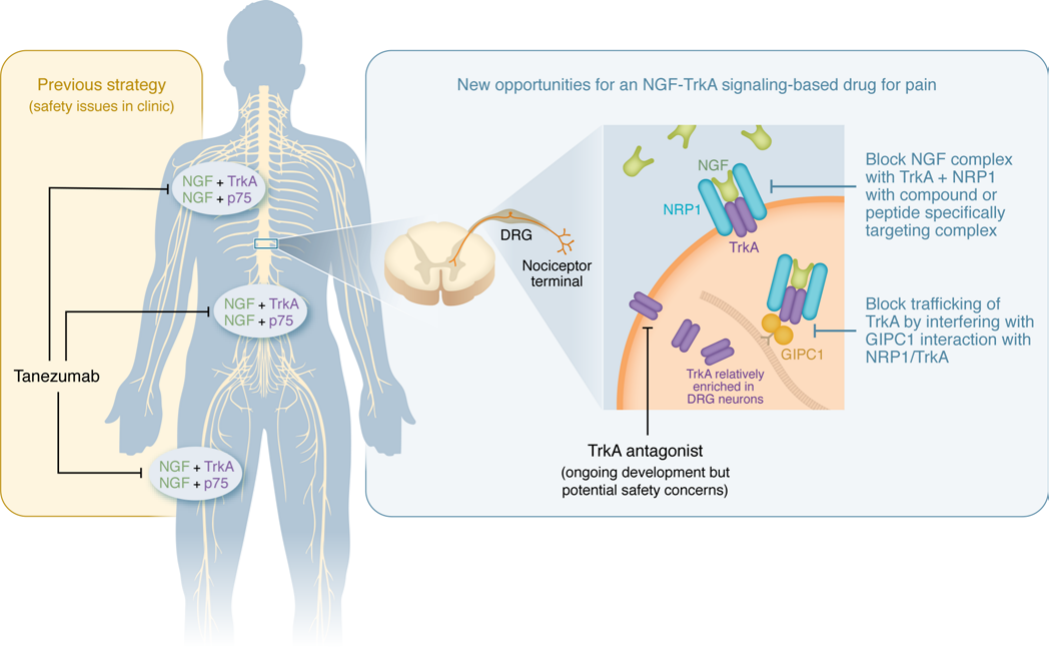
TrkA/NGF/NRP1/GIPC1 targeting for pain treatment. The findings of Peach et al. demonstrate that interfering with the TrkA/NGF/NRP1 protein complex, or with GIPC1-mediated assembly and/or trafficking of this complex influences NGF-driven pain. Because many of the genes involved in this signaling pathway are ubiquitously expressed, developing safe therapeutics for this pathway remains a challenge. Previous strategies such as tanezumab blocks NGF actions everywhere and have had safety issues. Similarly, targeting NRP1, GIPC1, NGF, and p75 to some extent, would also have an effect in all tissues expressed. Notably, the TrkA/NGF/NRP1 protein complex can impart specificity because the intersection of the expression and function of these proteins is relatively specific for DRG neurons and their axons, which innervate peripheral tissues.

Peach et al. provide compelling evidence that NRP1 serves as a crucial coreceptor for NGF and TrkA in pain signaling (13). Traditionally recognized for its roles in axon guidance and VEGF signaling (14), NRP1 was revealed to play a similar role in NGF-mediated pathways. Through microscale thermophoresis and bioluminescence resonance energy transfer (BRET) assays, Peach and colleagues demonstrated NGF binding to NRP1 with low nanomolar affinity. Molecular modeling further suggested a ternary NGF/TrkA/NRP1 complex with a 2:2:2 stoichiometry. NRP1 interacted with both NGF and TrkA, stabilizing their binding and enhancing signal transduction, demonstrating a very unexpected insight into NGF action at TrkA. Importantly, the study identifies a conserved R/KXXR/K motif in NGF’s C-terminus as critical for NRP1 binding, providing a basis for designing targeted inhibitors. The authors then showed that NRP1 and TrkA were coexpressed in nociceptive neurons in mice and human DRG (Figure 1). Immunohistochemistry and in situ hybridization revealed substantial overlap in calcitonin gene-related peptide–positive (CGRP-positive) (59%) and purinergic ionotropic receptor type 2X3–positive (P2X3-positive) (42%) neurons in human, indicating that these populations were primary mediators of NGF-induced pain. This precise cellular localization underscores NRP1’s interaction with TrkA’s relevance as a therapeutic target for pain modulation. Electrophysiology experiments showed that NRP1 inhibitors can suppress NGF-induced TRPV1-dependent ion channel activation in mouse DRG.

The study also identified GIPC1, an intracellular adaptor protein, as a key mediator of NRP1’s coreceptor function. GIPC1 scaffolds NRP1/TrkA interactions, coupling them to the myosin VI motor protein for trafficking to the membrane. Experimental inhibition of GIPC1 through siRNA reduced NGF-induced nociceptor excitation and pain-like behaviors in mice. These findings suggest that GIPC1 may offer an additional therapeutic target, further refining interventions aimed at the NGF/TrkA signaling cascade.

## Therapeutic implications

The discovery of NRP1’s role in NGF signaling represents an exciting therapeutic opportunity for chronic pain management. By targeting NRP1 and GIPC1, it may be possible to selectively modulate TrkA-mediated pain pathways while preserving NGF’s protective roles in other tissues whether those are mediated by TrkA or p75. This approach could avoid the side effect of rapidly progressing osteoarthritis that was observed in tanezumab trials (8) and ultimately led to the decision not to approve the therapeutic for osteoarthritis. As shown in the work by Peach and colleagues (13), NRP1 inhibitors demonstrated robust efficacy in preclinical models, suppressing NGF-induced TRPV1 sensitization, ionic currents, and nociception. For instance, the small-molecule NRP1 inhibitor EG00229 effectively blocked NGF-induced hyperexcitability in mouse and human nociceptors, supporting its potential for clinical translation (13). Unlike systemic NGF antibodies, NRP1-targeted therapies are less likely to interfere with NGF’s broader physiological roles. This specificity could address the long-term safety concerns that have hindered the approval of NGF-targeted treatments.

Looking ahead, several key questions and challenges must be addressed. While preclinical data are promising, additional rigorous preclinical experiments are needed to more fully understand this therapeutic opportunity. Particular attention should be given to evaluating the long-term effects of any potential NRP1/TrkA/NGF targeting therapeutic on joint health and NGF functionality in other tissues. However, long-term effects may be challenging to assess because NGF-sequestering therapeutics do not appear to readily cause rapidly progressing osteoarthritis, at least in dogs where bedinvetmab has been in clinical use for some time (15). Moreover, further studies are needed to delineate the precise molecular interactions within the NGF/TrkA/NRP1/GIPC1 axis. Structural analysis of the proposed ternary complex could inform the design of new inhibitors with enhanced specificity. This analysis is likely to be critical for drug design, as NRP1 and GIPC1 are widely expressed in human tissues and it is ultimately the interaction with TrkA that offers the specificity of targeting nociceptor NGF signaling (9) (Figure 1). It is also worth the effort to look beyond NRP1 and GIPC1, to search for other cofactors and downstream effectors of the NGF signaling cascade. Identifying such targets could offer additional targets for therapeutic development, and the work of Peach and colleagues gives insight into starting points for additional regulators of NGF/TrkA signaling in nociceptors.

## Conclusion

Our hope is that the work of Peach and colleagues will reinvigorate drug discovery and development work around NGF/TrkA targeting for pain. The problems emerging around the late-stage trials with tanezumab were a disappointment for the field of pain therapeutic development, but this pathway is still one of the most promising for nonopioid drugs for pain. In this case, the lessons from the first failures may open the door for innovative ideas, like those presented Peach et al. (13), that will ultimately lead to drugs that can benefit patients in need of new treatments for chronic pain.

## Version 1 02/17/2025

Electronic publication
